# Core neuromuscular co-activation mechanisms partly explain trunk kinematics in a perturbed postural task

**DOI:** 10.3389/fspor.2026.1716574

**Published:** 2026-03-13

**Authors:** Youri Duchene, Guillaume Mornieux, Arthur Petel, Philippe P. Perrin, Gérome C. Gauchard

**Affiliations:** 1 Université de Lorraine, DevAH - Development, Adaptation, Handicap, Nancy, France; 2 Université de Lorraine, CARE, Nancy, France; 3 Université de Lorraine, Faculty of Sport Sciences, Nancy, France; 4 University Hospital of Nancy, Laboratory for the Analysis of Posture, Equilibrium and Motor Function (LAPEM), Vandoeuvre–lès–Nancy, France

**Keywords:** core stability, EMG, ground translation, motor control, postural control

## Abstract

**Introduction:**

When facing a ground translation, trunk neuromuscular control remains unclear, despite its importance in sport practice, because core neuromuscular co-activations have not been investigated. Indeed, the spinal stiffness and trunk kinematics are impacted by core co-activation in dynamic tasks. Therefore, the aim of this study was to determine whether core neuromuscular co-activations could explain its motion during perturbed postural tasks.

**Methods:**

Thirty-six athletes (handball, karate, long jump) performed a perturbed postural task, facing six medial ground translations in unipedal stance. Trunk and hip kinematics were recorded with inertial measurement units and rectus abdominis, external obliques and erector spinal were measured with surface electromyography (EMG). Trunk and hip joint angles, as well as directed co-activation ratio and co-activation index between left and right EMG signals, were computed from 100 ms prior to the start of the translation until 800 ms after. Moreover, core neuromuscular latencies and trunk amplitude were calculated.

**Results:**

Core muscles co-activation ratios varied according to trunk kinematics during the perturbation. Furthermore, earlier activation for agonists' muscles combined with larger activation compared to antagonists' muscles in the early phase of the perturbation were noticed (*p* < 0.05). Lastly, co-activation ratios might partially predict trunk amplitude (R² between 19.3% to 39.1%) when participants were facing a medial translation on single leg stance. Early lateral trunk flexions could be considered as active control mechanisms ensuring postural stability.

**Conclusions:**

Overall, our results supported the active role of the core to maintain postural balance and underlined that the neuromuscular co-activation analysis is useful to provide a better understanding of core control.

## 1 Introduction

Maintaining body stability after a balance perturbation is a complex process. The lower limb kinematics and neuromuscular reactions to a postural perturbation have been widely documented in the literature, as they contribute to trunk position and therefore to center of mass (CoM) control [see Tokur et al., 2020 (1), for review]. However, while lower limb responses are crucial, the trunk itself plays a predominant role in postural control (2, 3), partly due to its large inertia (4). When facing a lateral ground translation in bipedal stance, minimal postural imbalance occurs, resulting in limited trunk and CoM motion (5). More challenging postural conditions have been introduced by reducing stance surface (5) or by having participants perform perturbed one-leg stance tasks (6, 7) to better understand the mechanisms of postural imbalance. For instance, a greater trunk lateral displacement was observed, measured at 8–10° in a narrow stance and 2° in a wide stance (5). During sport practice, large trunk lateral displacement, has been related to ACL injury risk (8, 9). Moreover, the ability to have an effective core stability is related to better sport performance (10) and postural control (11). Therefore, core neuromuscular control during perturbated postural tasks should be known to adapt core training protocols.

After a postural perturbation, most of the core neuromuscular analyses focused on activation latencies of agonists (i.e., muscles involved in trunk flexion, in the opposite direction of ground translation) and antagonists' muscles, as well as on the isolated muscle activation's amplitudes (12–14). The earlier agonists' activation compared to antagonists supported the hypothesis of an initial active phase that generates joint torques to move the CoM in the opposite direction of the ground translation, followed by a second phase correcting posture to maintain stability (12, 13). However, it also has been hypothesized that the trunk's inertia and lower limb torque production might be the main reason for a passive early trunk lateral lean (14). Therefore, investigating only isolated muscle activations might not fully describe the complexity of core neuromuscular mechanisms. Indeed, the co- activation between core muscles, increasing spine stiffness (15, 16), combined with concentric and eccentric modes of contraction (17), might serve as a postural control strategy to maintain balance. The co-activation analysis might be complementary to determine an active (i.e., early and larger agonists activations), a passive (i.e., later and smaller agonists activation) or a mixed pattern (e.g., balanced activation to stiffen the spine after an early agonists activation). In addition, the neuromuscular co-activation mechanisms in anticipation of a force applied to the upper trunk seem to be a strategy when the instability is high in the frontal plane (18) but not in the sagittal plane (19). Furthermore, co-activation ratios appear to vary according to trunk motion and dynamics (20–22), and help the understanding of joint motion during dynamic tasks (22–25). Therefore, core co-activations may help explain trunk motion during ground translations and could be combined with muscle latencies, as described above (12, 13), to depict more accurately trunk postural control mechanisms.

Hence, the main objective of the present study was to evaluate core neuromuscular control using co-activation ratios during perturbed balance tasks to provide a better understanding of postural and trunk control mechanisms. We hypothesized that (i) core muscles co-activation ratios would vary according to trunk kinematics before and during the postural perturbation, (ii) early lateral flexions would represent active control mechanisms and (iii) co-activation ratios would predict trunk kinematics accordingly.

## 2 Materials and methods

### 2.1 Participants

Thirty-six healthy male athletes participated in the study (mean (standard deviation (SD)) age: 23.3 (5.0); height: 1.83 (0.07) m; body weight: 79.4 (10.1) kg). This sample size was estimated on the basis of a power analysis to achieve 80% power at an alpha criterion level of 0.05 during linear multiple regressions with six predictors. Only males were recruited as the core stability could differ between sexes (8, 26, 27). This study is also part of a broader project that was restricted to male participants. All participants attended at least 3 training sessions a week and had over 10 years of experience in their respective sporting disciplines (handball, karate, and long jump). We chose these three sporting disciplines in order to study a broad range of postural control and footwork abilities to insure generalizable results to athletic male population. All participants were required to be free of lower limb or trunk injuries for the previous two months. All participants gave their written informed consent prior to participation. The study was approved by the institutional review board (Comité de Protection des Personnes Sud Méditerranée III; reference: 2018.07.03 bis).

### 2.2 Testing procedures

**Figure 1:**
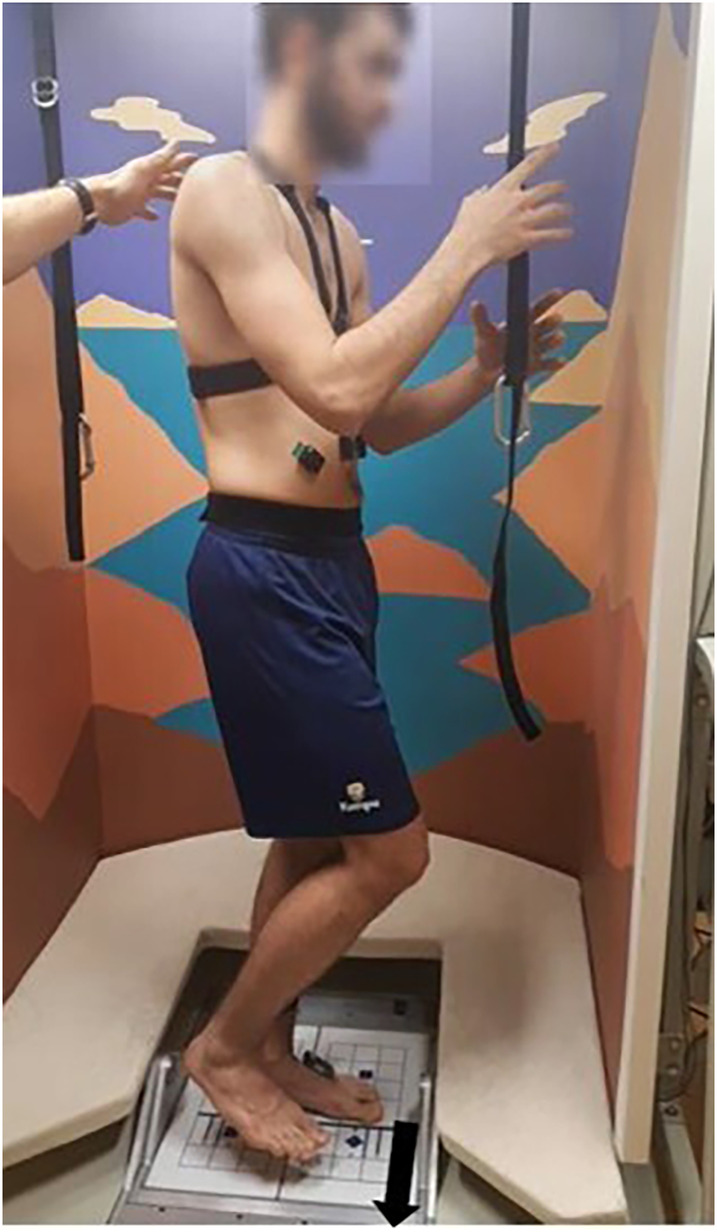
Participants position on the computerized dynamic posturography platform (Equitest®, Neurocom, Clackamas, OR, USA). The black arrow indicates the direction of the platform translation.

Participants performed six trials (barefoot), facing a medial ground translation while standing on their left leg (Figure 1). This task was chosen to induce the highest instability within frontal plane. Participants were asked to lift their right leg between 5 and 10 cm to feel comfortable and stable before the translation, with the hands at the shoulder level, close to a strap to avoid falls. Ground translation distance was height dependent and calculated as follows: 2.25 * height (in cm)/72. For a 1.83 m athlete, the translation distance is 5.7 cm. The duration was fixed and it lasted 550 ms. The maximal speed was 20 cm.s-1 and acceleration peak was 304.8 cm.s-2 (28).

### 2.3 Material

#### 2.3.1 Surface electromyography (EMG)

Bipolar surface electromyographic activity (EMG) was recorded using a 27 × 37 × 13 mm Avanti sensor, with a 10 mm inter-electrode spacing to reduce crosstalk contamination (29) (CMRR >80 dB, input impedance<0.75 µV; Trigno™, Delsys, Natick, MA, USA). The skin was shaved, slightly sanded and cleaned with an alcohol wipe and allowed to dry completely before the sensor fixation, ensuring a baseline at rest below 5 μV (inspected before recordings on EMGworks® software). Electrodes were attached parallel to the muscle fibers over the following muscles, bilaterally: the rectus abdominis (RA), the external obliquus (EO) and the longissimus erector spinae (ES), in line with the Surface Electromyography for the Non-Invasive Assessment of Muscles (SENIAM) guidelines (30). Therefore, RA electrodes were placed 2 cm laterally from the umbilicus, EO electrodes were placed at 15 cm laterally from the umbilicus and ES electrodes were placed about 2 cm lateral to the L1 spinous process. Data were collected at a sampling rate of 2000 Hz.

#### 2.3.2 Inertial measurement units (IMU)

The trunk (relative to the pelvis) and hip kinematics were measured using inertial measurement units (IMUs; CAPTIV, Tech Ergo Appliquées, Vandoeuvre-lès-Nancy, France). The IMUs were placed laterally on the middle of the left thigh, between the posterior superior iliac spines, and on the sixth thoracic vertebra. Before the static trial, the magnetometer reading was checked and set to zero with the participant in quiet stance. The sensors were consistently position so the x, y and z axes of the sensors follow respectively the medio-lateral, top-bottom, and anterior-posterior segments axes. A static calibration was performed. Data were collected at a sampling rate of 64 Hz. Quaternions were converted to angles using the CAPTIV system software. The system featured a resolution of 0.1°.

#### 2.3.3 Ground support

The moving platform (EquiTest®, Neurocom, Clackamas, OR, USA) was equipped with an accelerometer (Trigno™, Delsys, Natick, MA, USA) on a corner, synchronized with EMG sensors and IMUs, to detect the start and the end of each ground translation. All signals were synchronized digitally by the Captiv system.

### 2.4 Variables

EMG data were band-pass filtered between 10 Hz to 500 Hz using a 4th order Butterworth bandpass filter. For the RA and EO signals, the heart's electrical activity was filtered out using a 4th order 30 Hz Butterworth high-pass filter (31). Root mean square (RMS) EMG amplitudes were computed over 100 ms windows, spanning from 100 ms before the perturbation (PRE) to 800 ms after. A 25 ms electromechanical delay was considered only for the RMS calculation, applied as a backward temporal shift (32). RMS values were normalized against the RMS value recorded during a 40s bipedal static trial. The latency between the start of the perturbation and the beginning neuromuscular activity was defined as the first activation peak exceeding 3 SD above the baseline (33, 34). A visual inspection was done by the same experimenter (YD) on each trial to ensure the quality of the signal (34).

The simultaneous activity of agonist and antagonist muscles was analyzed via directed co-contraction ratios (DCCR) (24) and co-contraction indexes (CCI) (35) during each 100 ms window. DCCR and CCI respectively reflected the balance between agonist and antagonist muscles activations and the intensity of co-activation. These ratios were defined according to the anatomical function of the muscles with respect to motion during the perturbation. DCCR values ranged from −1 (towards antagonists) to 1 (towards agonists), while a value of zero would indicate equal mean activation of agonist and antagonist muscle groups (Equation 1). A positive ratio would indicate greater mean co-activation of left (i.e., lateral) RA, EO and ES muscles (agonists) than right (i.e., medial) muscles (antagonists). Agonists and antagonists mean activation were compared and defined as lower and higher EMG depending on their level of activation to calculate CCI (Equation 2). The CCI value during quiet stance is 200, reflecting the low intensity of co-activation during this task. Both ratios are without units.

Equation 1. Directed co-contraction ratio (DCCR) computation.

If the mean agonist activation is higher than mean antagonist activation:

$$\mathrm{DCCR}=1\text{-}\frac{\bar{\mathrm{EMG}\;\mathrm{of}\;\mathrm{antagonist}\;\mathrm{muscles}}}{\bar{\mathrm{EMG}\;\mathrm{of}\;\mathrm{agonist}\;\mathrm{muscles}}}$$

Else:

$$\mathrm{DCCR}=\frac{\bar{\mathrm{EMG}\;\mathrm{of}\;\mathrm{agonist}\;\mathrm{muscles}}}{\bar{\mathrm{EMG}\;\mathrm{of}\;\mathrm{antagonist}\;\mathrm{muscles}}}\text{-}1$$

Equation 2. Co-contraction index (CCI) computation. Lower and higher EMG are defined by comparing the mean values of the agonist's activation compared to the antagonist's activation.

$$\mathrm{CCI}=\frac{\mathrm{lower}\;\bar{\mathrm{EMG}}}{\mathrm{higher}\;\bar{\mathrm{EMG}}}*\;(\mathrm{lower}\;\bar{\mathrm{EMG}}+\mathrm{higher}\;\bar{\mathrm{EMG}})$$

Joint angles were computed for the trunk (relative to the pelvis) and the hip. Based on residual analysis, kinematic data were filtered using a 4th order 15 Hz low-pass Butterworth filter. Trunk amplitude from the start of the perturbation to its maximal angular value and the time taken to reach this maximum value were assessed. Moreover, angular displacement for each 100 ms windows from the start to 800 ms post-perturbation were calculated.

### 2.5 Statistical methods

Means and SDs were calculated for all measured variables across the six trials for each participant. Normality of distribution and variance homogeneity were respectively verified via Lilliefors' and Levene's tests. A repeated-measures ANOVA was conducted for each condition and each variable to assess the main effect of perturbation time. Sphericity was assessed using Mauchly's test. When violated, Greenhouse–Geisser corrections were applied. In case of a significant main effect, paired t-tests with a Bonferroni correction were used to examine differences between each subsequent 100 ms window. Student's t-tests were performed to compare EMG latencies between the different muscles. To determine the relationship between co-activation variables and kinematics, two multiple linear regressions were performed, one with DCCR (regression model 1) and the other with CCI (regression model 2), to predict trunk lateral amplitude. To check for multicollinearity, we examined the variance inflation factor (VIF) for each regression model with a threshold of 5.0. After 500 ms, the neuromuscular ratio variables were impacted by multicollinearity. Therefore, the five 100 ms windows from PRE (−100 ms) to 400 ms were inserted as predictors in the models. Statistical analyses were conducted using Statistica software (version 13; StatSoft, Inc., Tulsa, OK). The threshold for statistical significance was set to *p* < 0.05.

## 3 Results

### 3.1 Kinematics

**Figure 2:**
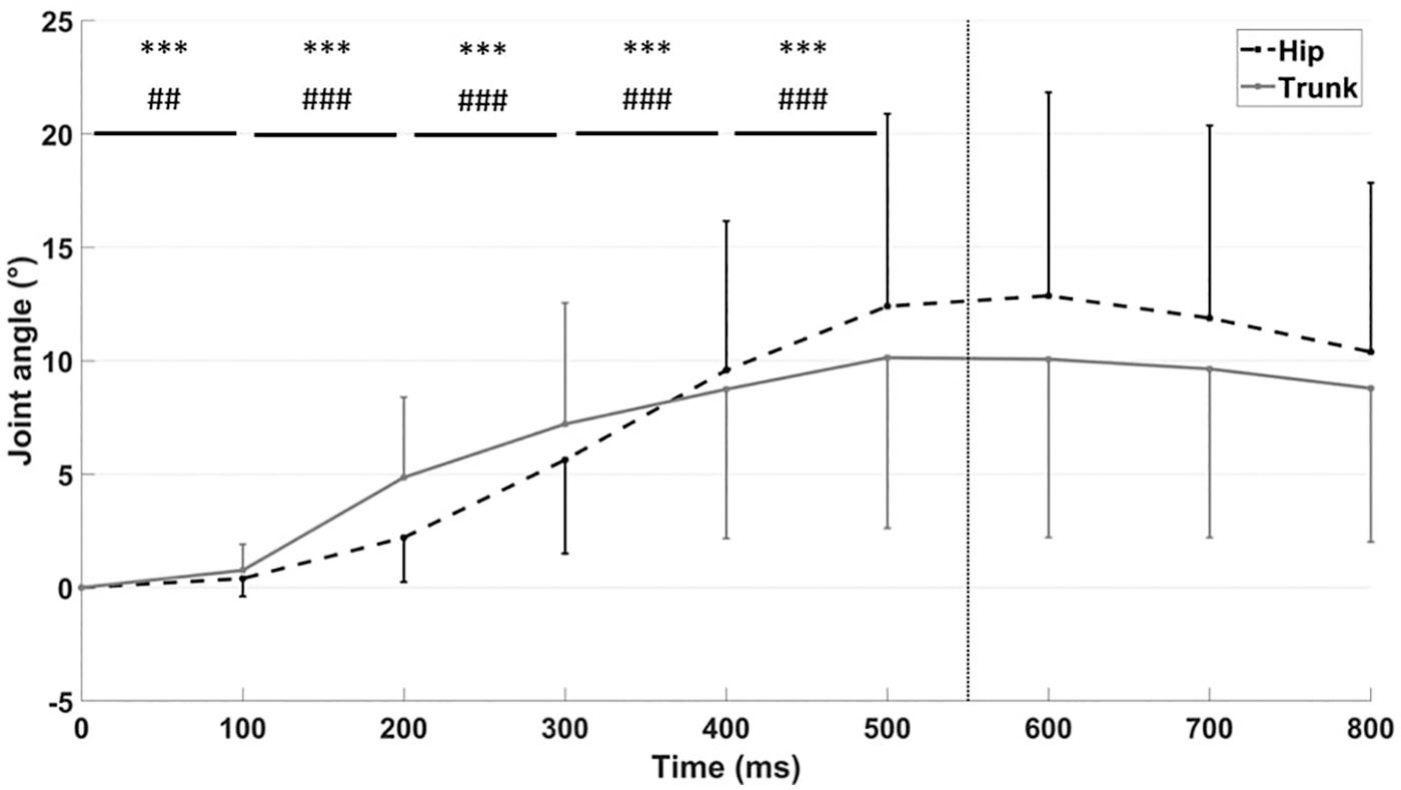
Trunk and hip angle variations in the frontal plane (°) from the start of the perturbation (0 ms) to 800 ms with 100 ms windows. The vertical dashed bar represents the end of the perturbation (550 ms). Positive values express lateral flexion. ***: indicates a significant difference between two subsequent 100 ms intervals for the trunk (*p* < 0.001). ###: indicates a significant difference between two subsequent 100 ms intervals for the hip (*p* < 0.001).

Lateral lean of the hip and the trunk increased significantly (F(7, 245) = 39.6, *p* < 0.001, *η*^2^*p* = 0.53 and F(7, 245) = 27.7, *p* < 0.001, *η*^2^*p* = 0.44 respectively) during the first 500 ms before stabilization (Figure 2). Trunk angular peak (11.9° ± 7.8°) occurred at 571 ± 183 ms after the start of the perturbation.

### 3.2 EMG

**Figure 3:**
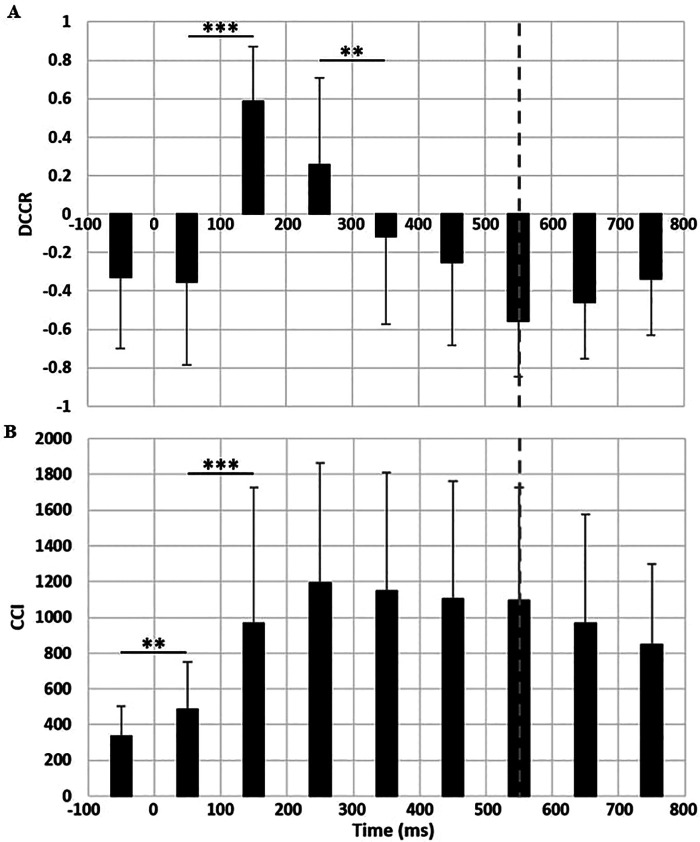
**(A)** directed co-contraction ratio (DCCR) and **(B)** co-contraction index (CCI) between lateral and medial muscles. Perturbation begins at 0 ms and ends at 550 ms (vertical dashed bar). *: indicates a significant difference between two subsequent 100 ms intervals (*p* < 0.05).

DCCR were mainly orientated towards medial activation (negative values), indicating higher activations for medial (right) muscles than lateral (left) muscles, excepted between 100 ms and 300 ms (Figure 3A). Repeated-measure ANOVA was significant (F(8,280) = 51.0, *p* < 0.001, *η*^2^*p* = 0.59) and *post-hoc* significance is presented on Figure 3A. CCI were relatively low before the perturbation with respect to maximal values reached during the perturbation (CCI_PRE=336 ± 169 vs. CCI_300 = 1,194 ± 668). For the CCI, the ANOVA was significant (F(8,280) = 17.4, *p* < 0.001, *η*^2^*p* = 0.33). A significant increase in CCI occurred until 200 ms, followed by a plateau representing a high level of co-activation maintained until the end of the perturbation (Figure 3B).

**Figure 4:**
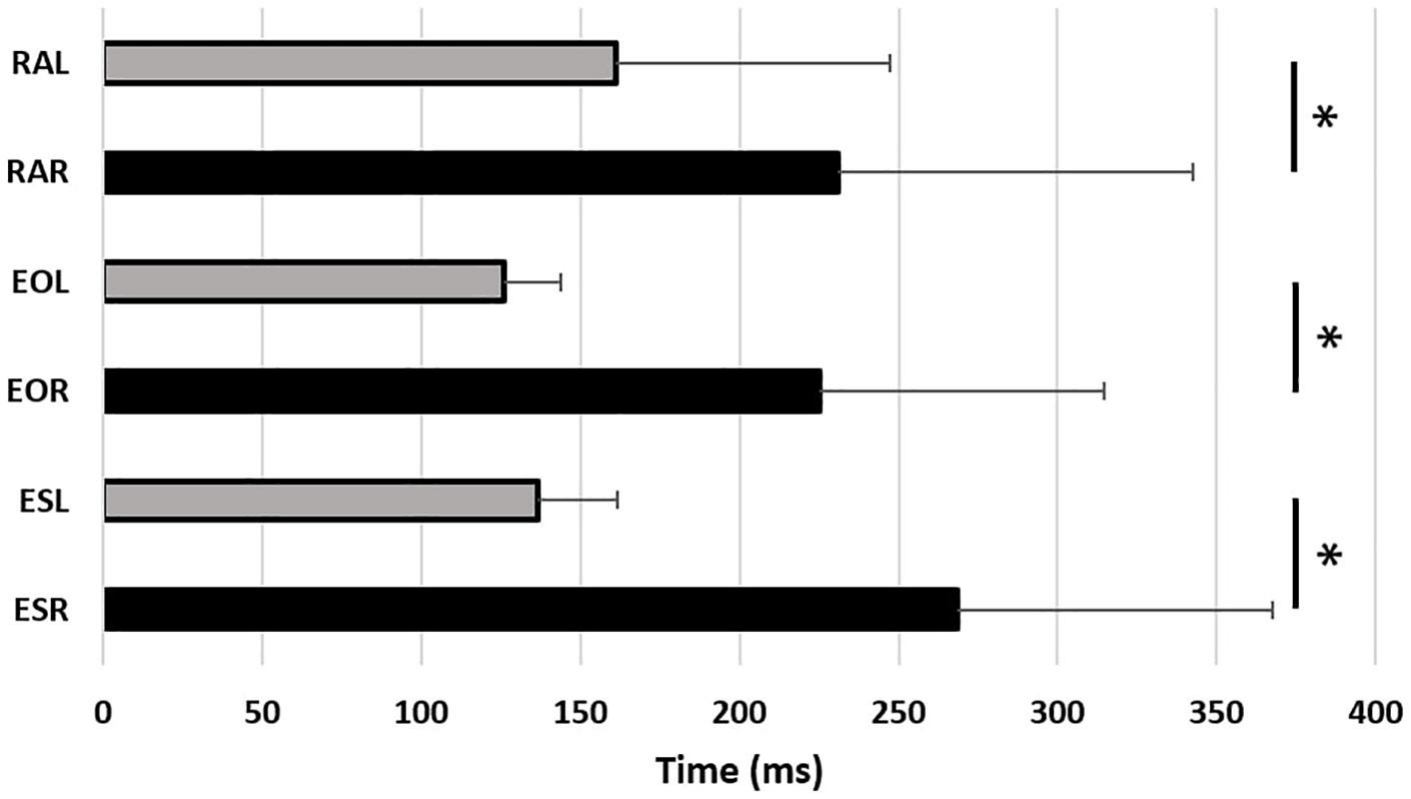
Trunk muscles activation latencies (ms) after the perturbation (0 ms) for the rectus abdominis left (RAL) and right (RAR), the external obliques left (EOL) and right (EOR) and the erector spinae left (ESL) and right (ESR). *: indicates a significant difference between muscles (*p* < 0.05).

Moreover, left muscles (lateral) activations occurred earlier than right ones (medial) (*p* < 0.05) (Figure 4).

### 3.3 Regression models

For model 1, DCCR predicted 39.1% of trunk lateral amplitude variance (adjusted R^2^ = 0.391, *p* < 0.01). Significant *β*-coefficients revealed that higher DCCR were correlated with less trunk lateral amplitude before the translation (*β*=−0.48), but with larger trunk amplitude during the 300–400 ms window (*β*=0.62) and in a lesser extend a tendency was noted between 100 and 200 ms (*β*=0.26, *p* < 0.07).

In the model 2, CCI predicted 19.3% of this variance (adjusted R^2^ = 0.193, *p* < 0.05). Higher CCI was correlated to a larger trunk lateral amplitude during the 300–400 ms window (*β*=0.79) and tended to limit the trunk amplitude between 100 and 200 ms (*β*=−0.3, *p* < 0.07).

## 4 Discussion

The present study aimed to investigate whether neuromuscular co-activation ratios could core contribution to postural and trunk control during a perturbed postural task. The main findings were (i) that core muscles co- activation ratios varied according to trunk kinematics before and during the perturbation, (ii) that early lateral trunk neuromuscular activation, as well as an increase of DCCR were noticed, and (iii) that co-activation ratios partially (between 21.9% and 39.7%) predicted trunk amplitude during medial translation on single leg stance.

Prior to the translation, the DCCR was negative due to the specificity of the single leg stance. Indeed, medial core muscles might require higher activation to avoid pelvic drop to maintain postural stability. A complementary hypothesis might be that participants increased their medial activations in anticipation of the perturbation. Then, between 100 ms and 300 ms, DCCR switched to positive values, indicating a higher activation of lateral muscles, combined with a large increase in trunk lateral angle. These results, combined with the shorter latencies for lateral muscles noted in the present study, suggest that the trunk lateral lean following the perturbation is an active control mechanism, and not only the passive response to the ground translation (14). This early strategy to tilt the trunk laterally could help countering center of mass large medial acceleration. Overall, the larger CCI, theoretically related to greater spinal stiffness (16), combined with greater medial muscle activation, probably helps to stabilize the trunk by reducing its angle variation and then by stabilizing its position. This behavior might also help to medially accelerate the center of mass to return to the initial position (36).

Moreover, lateral DCCR explained 39.7% of the variance in trunk lateral amplitude, indicating a modest but significant contribution on frontal trunk control. Prior to ground translation, increasing medial activation before the perturbation did not limit trunk amplitude, but rather increased it. Indeed, overcompensation by anticipatory postural adjustments can increase the actual postural perturbation (19). With the DCCR oriented laterally between 100 and 200 ms, the analysis of the *β* coefficient highlighted that a concentric action of lateral muscles impacted the frontal angular displacement of the trunk. Between 300 and 400 ms, the DCCR was oriented medially, suggesting an eccentric control of these muscles to limit lateral trunk inclination. CCI explained only 21.9% of the variance in trunk lateral amplitude, making it a less functional variable to explain trunk kinematics. However, the analysis of the *β*-coefficients revealed a tendency for increasing the intensity of co-activations between 100 and 200 ms to limit trunk inclination. Surprisingly, from 300 ms onwards, an increase in CCI was linked to greater trunk inclination. As the statistical model was based on correlations, we can assume that the intensity of co-activations might be dependent on trunk displacement. Indeed, a greater trunk inclination would require higher levels of co-activations to stabilize the spine.

Furthermore, our results do not provide clear evidence supporting the existence of a major anticipatory co-activation mechanism in anticipation of a perturbation, as suggested by Santos & Aruin (2009) (18). Another hypothesis, developed by Aruin et al. (1998) (19) in the context of an upper-body perturbation with ground instability, is that smaller anticipatory postural adjustments could avoid generating additional imbalances prior to the perturbation, compared to a stable ground condition. This interpretation might be consistent with the relatively low level of co-activation observed before perturbation onset in the present study, despite differences in the perturbation paradigm. This hypothesis is also consistent with the work of Vera-Garcia et al. (2007) (37), which demonstrated that voluntarily increasing co-activations in advance did not lead to a better control of core stability during isolated trunk sudden loading tasks, which differs from our postural task. This suggests that investigating this hypothesis in future studies could help to understand better the adequate neuromuscular activations needed to insure stability in different challenging postures.

We acknowledge the limitation that the perturbation's fixed duration can induce some variability between tall and short participants by modifying the peak velocity. However, a height-dependent duration would delay the timing of the peak velocity and could also induce variability between participants. Finally, our results indicated that neuromuscular core stability programs should consider incorporating perturbation-based exercises to better prepare athletes for the demands of combat and team sports. In particular, ground perturbation training has been shown to improve lower-limb kinematics and neuromuscular co-activation patterns associated with ACL loading (38). Given that excessive trunk lateral lean is a key factor contributing to ACL injury risk (9), and that the lateral lean observed during sidestepping tasks also appears to reflect an active control strategy (25), targeted training of this capacity may enhance trunk control during sidestepping tasks although its direct impact on ACL injury risk remains to be established.

## 5 Conclusion

The novelty of the present findings lies in three main aspects. First, co-activation mechanisms seem to vary according to trunk kinematics. Also, the larger and earlier activation of lateral muscles supports that trunk inclination in the opposite direction of a medial perturbation suggests an active mechanism of postural control, controlling external forces and trunk's inertia. Then, co-activations are not a major mechanism used in anticipation of perturbation, but greater emphasis is placed on muscles antagonistic to trunk displacement than to agonists prior to perturbation. Overall, this work provides novel insight into the relationship between core neuromuscular co-activation and trunk kinematics, thereby enhancing our understanding of core motor control during perturbed postural tasks. On a more forward-looking level, these neuromuscular co-activation mechanisms would play a major role during dynamic, sport-specific tasks. This enhanced understanding of core stability might facilitate the development of refined neuromuscular training aiming to improve trunk and postural control, potentially leading to optimized performance and injury prevention in sports contexts.

## Data Availability

The raw data supporting the conclusions of this article will be made available by the authors, without undue reservation.

## References

[1] Tokur D Grimmer M Seyfarth A. Review of balance recovery in response to external perturbations during daily activities. Hum Mov Sci. (2020) 69:102546. 10.1016/j.humov.2019.102546 31989948

[2] Kilby MC Molenaar PCM Newell KM. Models of postural control: shared variance in joint and COM motions. PLoS One. (2015) 10(5). 10.1371/journal.pone.0126379 25973896 PMC4431684

[3] Duchene Y Mornieux G Petel A Perrin PP Gauchard GC. The trunk’s contribution to postural control under challenging balance conditions. Gait Posture. (2021) 84:102–7. 10.1016/j.gaitpost.2020.11.020 33290903

[4] Massion J. Movement, posture and equilibrium: interaction and coordination. Prog Neurobiol. (1992) 38(1):35–56. 10.1016/0301-0082(92)90034-C 1736324

[5] Henry SM Fung J Horak FB. Effect of stance width on multidirectional postural responses. J Neurophysiol. (2001) 85(2):559–70. 10.1152/jn.2001.85.2.559 11160493

[6] Pintsaar A Brynhildsen J Tropp H. Postural corrections after standardised perturbations of single limb stance: effect of training and orthotic devices in patients with ankle instability. Br J Sports Med. (1996) 30(2):151–5. 10.1136/bjsm.30.2.151 8799602 PMC1332381

[7] Wang Z Molenaar PCM Challis JH Jordan K Newell KM. Visual information and multi-joint coordination patterns in one-leg stance. Gait Posture. (2014) 39(3):909–14. 10.1016/j.gaitpost.2013.11.017 24388780

[8] Zazulak BT Hewett TE Reeves NP Goldberg B Cholewicki J. Deficits in neuromuscular control of the trunk predict knee injury risk: prospective biomechanical-epidemiologic study. Am J Sports Med. (2007) 35(7):1123–30. 10.1177/0363546507301585 17468378

[9] Hewett TE Myer GD. The mechanistic connection between the trunk, hip, knee, and anterior cruciate ligament injury. Exerc Sport Sci Rev. (2011) 39(4):4. 10.1097/JES.0b013e3182297439 21799427 PMC4168968

[10] Hibbs AE Thompson KG French D Wrigley A Spears I. Optimizing performance by improving core stability and core strength. Sports Med. (2008) 38(12):12. 10.2165/00007256-200838120-00004 19026017

[11] Cabrejas C Morales J Solana-Tramunt M Nieto-Guisado A Badiola-Zabala A Campos-Rius J. Does 8 weeks of integrated functional core and plyometric training improve postural control performance in young rhythmic gymnasts? Motor Control. (2022) 1:1–23. 10.1123/mc.2022-0046 35894881

[12] Henry SM Fung J Horak FB. Control of stance during lateral and anterior/posterior surface translations. IEEE Trans Rehabil Eng. (1998) 6(1):32–42. 10.1109/86.662618 9535521

[13] Henry SM Fung J Horak FB. EMG Responses to maintain stance during multidirectional surface translations. J Neurophysiol. (1998) 80(4):1939–50. 10.1152/jn.1998.80.4.1939 9772251

[14] Gorgy O Vercher J-L Coyle T Franck B. Coordination of upper and lower body during balance recovery following a support translation. Percept Mot Skills. (2007) 105(3):715–32. 10.2466/pms.105.3.715-732 18229529

[15] McGill SM Cholewicki J. Biomechanical basis for stability: an explanation to enhance clinical utility. J Orthop Sports Phys Ther. (2001) 31(2):2. 10.2519/jospt.2001.31.2.96 11232744

[16] Lee PJ Rogers EL Granata KP. Active trunk stiffness increases with co-contraction. J Electromyogr Kinesiol. (2006) 16(1):51–7. 10.1016/j.jelekin.2005.06.006 16099678 PMC1635026

[17] Preuss R Fung J. Musculature and biomechanics of the trunk in the maintenance of upright posture. J Electromyogr Kinesiol. (2008) 18(5):815–28. 10.1016/j.jelekin.2007.03.003 17449280

[18] Santos MJ Aruin AS. Effects of lateral perturbations and changing stance conditions on anticipatory postural adjustment. J Electromyogr Kinesiol. (2009) 19(3):532–41. 10.1016/j.jelekin.2007.12.002 18249139

[19] Aruin AS Forrest WR Latash ML. Anticipatory postural adjustments in conditions of postural instability. Electroencephalogr Clin Neurophysiol. (1998) 109(4):350–9. 10.1016/S0924-980X(98)00029-0 9751298

[20] Marras WS Mirka GA. Muscle activities during asymmetric trunk angular accelerations. J Orthop Res. (1990) 8(6):824–32. 10.1002/jor.1100080607 2213339

[21] Marras WS Granata KP. Spine loading during trunk lateral bending motions. J Biomech. (1997) 30(7):697–703. 10.1016/S0021-9290(97)00010-9 9239549

[22] Duchene Y Simon FR Ertel GN Maciejewski H Gauchard GC Mornieux G. The stroke rate influences performance, technique and core stability during rowing ergometer. Sports Biomechanics. (2024) 24(6):1576–93. 10.1080/14763141.2024.2301992 38205960

[23] Jamison ST McNally MP Schmitt LC Chaudhari AMW. The effects of core muscle activation on dynamic trunk position and knee abduction moments: implications for ACL injury. J Biomech. (2013) 46(13):2236–41. 10.1016/j.jbiomech.2013.06.021 23891313

[24] Donnelly CJ Elliott BC Doyle TLA Finch CF Dempsey AR Lloyd DG. Changes in muscle activation following balance and technique training and a season of Australian football. J Sci Med Sport. (2015) 18(3):348–52. 10.1016/j.jsams.2014.04.012 24880917

[25] Duchene Y Gauchard GC Mornieux G. Influence of sidestepping expertise and core stability on knee joint loading during change of direction. J Sports Sci. (2022) 40(9):959–67. 10.1080/02640414.2022.2042980 35191363

[26] Kuniki M Iwamoto Y Kito N. Effects of core stability on shoulder and spine kinematics during upper limb elevation: a sex-specific analysis. Musculoskelet Sci Pract. (2022) 62:6. 10.1016/j.msksp.2022.102621 35926474

[27] Anderson BE Boyce KK Christian ME Bay RC Huxel Bliven KC. Relationships between core stability, sex, and movement capacity. Athletic Train Sports Health Care. (2020) 12(3):111–8. 10.3928/19425864-20190301-01

[28] Shepard NT Schultz A Gu MJ Alexander NB Boismier T. Postural control in young and elderly adults when stance is challenged: clinical versus laboratory measurements. Ann Otol Rhinol Laryngology. (1993) 102(7):508–17. 10.1177/000348949310200704 8333672

[29] De Luca CJ Kuznetsov M Gilmore LD Roy SH. Inter-electrode spacing of surface EMG sensors: reduction of crosstalk contamination during voluntary contractions. J Biomech. (2012) 45(3):555–61. 10.1016/j.jbiomech.2011.11.010 22169134

[30] Hermens HJ Freriks B Disselhorst-Klug C Rau G. Development of recommendations for SEMG sensors and sensor placement procedures. J Electromyogr Kinesiol. (2000) 10(5):361–74. 10.1016/S1050-6411(00)00027-4 11018445

[31] Drake JDM Callaghan JP. Elimination of electrocardiogram contamination from electromyogram signals: an evaluation of currently used removal techniques. J Electromyogr Kinesiol. (2006) 16(2):175–87. 10.1016/j.jelekin.2005.07.003 16139521

[32] Le Mansec Y Dorel S Nordez A Jubeau M. Is reaction time altered by mental or physical exertion? Eur J Appl Physiol. (2019) 119(6):1323–35. 10.1007/s00421-019-04124-7 30879187

[33] Hodges PW Bui BH. A comparison of computer-based methods for the determination of onset of muscle contraction using electromyography. Electroencephalogr Clin Neurophysiol. (1996) 101(6):511–9. 10.1016/S0921-884X(96)95190-5 9020824

[34] Allison GT. Trunk muscle onset detection technique for EMG signals with ECG artefact. J Electromyogr Kinesiol. (2003) 13(3):209–16. 10.1016/S1050-6411(03)00019-1 12706601

[35] Rudolph KS Axe MJ Snyder-Mackler L. Dynamic stability after ACL injury: who can hop? Knee Surg Sports Traumatol Arthrosc. (2000) 8(5):262–9. 10.1007/s001670000130 11061293

[36] Patla AE Adkin A Ballard T. Online steering: coordination and control of body center of mass, head and body reorientation. Exp Brain Res. (1999) 129(4):0629–34. 10.1007/s002210050932 10638436

[37] Vera-Garcia FJ Elvira JLL Brown SHM McGill SM. Effects of abdominal stabilization maneuvers on the control of spine motion and stability against sudden trunk perturbations. J Electromyogr Kinesiol. (2007) 17(5):556–67. 10.1016/j.jelekin.2006.07.004 16996278

[38] Hurd WJ Chmielewski TL Snyder-Mackler L. Perturbation-enhanced neuromuscular training alters muscle activity in female athletes. Knee Surg Sports Traumatol Arthrosc. (2006) 14(1):60–9. 10.1007/s00167-005-0624-y 15937713

